# Experiences and challenges of home care nurses and general practitioners in home-based palliative care – a qualitative study

**DOI:** 10.1186/s12904-018-0350-0

**Published:** 2018-07-18

**Authors:** Britt Viola Danielsen, Anne Marit Sand, Jan Henrik Rosland, Oddvar Førland

**Affiliations:** 1 Department of Health and Caring Sciences, Faculty of Health and Social Sciences, Western Norway University of Applied Sciences, P.O. Box 7030, N-5020 Bergen, Norway; 2 Department of Clinical Medicine, University of Bergen, Bergen, Norway; 3 Sunniva Centre for Palliative Care, Haraldsplass Deaconess Hospital Bergen, Bergen, Norway; 4 Centre for Care Research - Western Norway, Western Norway University of Applied Sciences, Bergen, Norway; 5 Faculty of Health Studies, VID Specialized University, Bergen, Norway

**Keywords:** Palliative home care, Home care nurse, General practitioner, Home deaths, Qualitative study

## Abstract

**Background:**

Norway has one of the lowest home death rates in Europe. However, it is the health authorities´ ambition to increase this by facilitating palliative care at home. The aim of this study was to achieve more insight, through home care nurses and general practitioners, of conditions that facilitate or hamper more time at home and more home deaths for patients with terminal disease and short life expectancy.

**Methods:**

We used a qualitative research design with four focus groups with a total of 19 participants, of either home care nurses or general practitioners, using semi-structured question guides. The data were processed by systematic text condensation and encompassed thematic analysis of meaning and content of data across cases, which included four steps of analysis.

**Results:**

Three main themes were identified: 1) The importance of a good start for the patient and family with five sub-themes, 2) ‘Passing the baton’ – the importance of collaboration across the health system with four sub-themes, and 3) Avoiding new hospitalization by establishing collaboration and competence within primary health care with four sub-themes.

**Conclusions:**

This study demonstrates that optimum palliative care at home depends on close collaboration and dialogue between the patient, family, home care nurses and general practitioner. It suggests the need for safer discharge routines and planning when hospitals transfer patients with terminal disease to their homes. A good start for the patient and family, where the initial interdisciplinary collaboration meeting takes place in the patient’s home, is crucial for a good result. The general practitioners’ perception of their ‘disconnection’ during hospitalization and prior to discharge has the potential to reduce patient safety. The family seems to be fundamental in gaining more time at home for the patient and supporting the patient to eventually die at home. Home-based palliative care demands experience and competence as well as regular supportive mentoring.

## Background

Norway has one of the lowest home death rates in Europe. Only 13% of deaths in Norway took place at home in 2016, while 50% died in a nursing home and 30% in a hospital [1]. In Norway, there is, so far, no trend towards an increasing proportion of home deaths, even though the health care authorities have tried to facilitate palliative care services in the home [2, 3]. In some countries, this trend has been reversed in recent decades, particularly in the United States, Canada and the United Kingdom [4–7], with greater proportions of people dying at home.

Dying at home surrounded by family members is considered by some to be the ‘gold standard’ for a good death [8–10]. Several systematic literature reviews found that most of those with terminal disease and short life expectancy wanted to stay in their homes as long as possible, and eventually die at home [11–13]. One systematic review from 2015 emphasized that many studies highlighted patients´ preferences to be cared for at home as long as possible or to be able to die at home. However, for some patients, dying at home may be difficult to achieve due to an absence of family caregivers [14]. Another systematic review from 2015 concluded that caution should be taken when assuming that most patients prefer to die at home, because studies often excluded the views of those who did not express a preference or who were not asked [15]. Despite this, it is important that health care services support those preferring to stay at home in the last part of their lives [16]. According to Norwegian health authorities, increased time at home can often be a more realistic objective for home-based palliative care than home death, as it says something about the extent to which the patient’s desire to live the last time at home is fulfilled [17].

In Norway, research undertaken in a large municipality concluded that a multidisciplinary approach, which involved family physicians and community nurses with support from a multidisciplinary specialist palliative care consultant team, increased the proportion of home deaths and led to fewer people being admitted to nursing homes [18]. In a systematic review, Murray et al. [11] concluded that factors related to the disease, the degree of social support from relatives, the quality and availability of healthcare services and programs, and the patient’s preference were the most important factors for determining the place of end-of-life care. This is in line with Gomes and Higginson [19], who found the place of death to be an interplay of factors such as type of disease and disability, sociodemographic characteristics, patients’ preferences, health care services, social support from the family, and macrosocial factors such as historical trends. A systematic review and meta-analysis of determinants of home and nursing home deaths from 2016 concluded that early referral to palliative care and the presence of a multidisciplinary home-based palliative care team increase the likelihood of a patient dying in their preferred location [8]. These studies identify the interplay between the patient, family, health care services, general practitioners (GPs) and home care nurses to be critical factors.

To further explore these aspects, interviewing health care personnel could be a method to achieve more precise insight and understanding. We found only a few qualitative studies of experiences with home-based palliative care that included both GPs and home care nurses. In an evaluation study from England, specialist palliative care nurses, district nurses and GPs stressed three elements of importance for keeping patients at home at the end of their life: firstly, an accompanied transfer home element, which could be arranged quickly; secondly, trained hospice aides at home to care and support patients and their families; and thirdly, a crisis intervention team that included a specialist palliative care physician [20]. Focus groups with district nurses and community specialist palliative care nurses in England identified poor co-ordination and discharge planning, difficulty in arranging necessary equipment and services and inadequate out-of-hours medical provision as barriers to dying at home for patients with cancer with a short life expectancy [21].

The aim of the current study was to achieve more insight, through home care nurses and GPs, of conditions that facilitate or hamper more time at home and more home deaths for patients with terminal disease and short life expectancy.

## Methods

### Study context

Norway has a national tax-funded, universal, public, long-term care service aimed at securing each patient’s basic medical and social needs, free of charge or with a small user fee. The service is primarily carried out at a local level in the municipalities through GP cooperatives, home care services, nursing homes and sheltered houses. The home care service is part of the municipality health care service, and includes home care nursing and practical assistance based on each patient’s needs [22].

Each Norwegian citizen has a right to have his or her own family doctor, a GP. To be a patient on a GP list is free of charge, and if you need to see the doctor, you have to pay a limited fee, the balance being paid by the state. The GP collaborates with the home care nursing services in the municipality. Home care nursing is a municipal, public service, whereas GPs are self-employed and organised in GP cooperatives. The GP identifies medical needs, gives prescriptions and carries out home visits if considered necessary [23].

Inpatient care is mainly provided by the hospital trusts owned by Regional Health Authorities and founded by the state. The largest hospitals have multidisciplinary palliative care teams providing care and sometimes ambulatory services to patients with the most complex needs in hospital departments and municipalities nearby [23]. Since the late 1980s, there has been a policy of replacing relatively expensive inpatient care with less-costly outpatient care and bringing care closer to patients’ homes. This policy has been strengthened by the Coordination Reform implemented in 2012 [24].

According to this policy, the provision of care in Norway has been shifting from institutional care towards more home-based care. The home care nurses play a fundamental role, including palliative care in the home. Home-based care can be provided as a 24-h service, giving health and care services at patients’ homes several times during both the day and night. Palliative care for patients staying at home is provided via collaboration between next of kin, home care nurses and GPs, and is sometimes assisted by a hospital-based specialist palliative care team [23, 25]. The GPs often have a history of providing health care for the palliative care patient and the family over many years and are often involved in the early stages of diagnosis, thereby providing some continuity of oversight. In our sample, they generally had one to two palliative care patients in home care each year for whom they had medical responsibility. Medical doctors are the only health professionals in Norway who can prescribe medicines, as nurse practitioners have not yet been introduced.

### Study design, participants and sampling populations

Our study had a qualitative approach with a phenomenological dimension. The use of focus groups is a qualitative research method, which is suitable for gaining rich descriptions of the participants’ experiences and in-depth knowledge about complex phenomena [26]. We conducted focus groups separately for home care nurses, nursing assistants and GPs, using a semi-structured guide for the research questions. The question guides were thoroughly discussed and refined in the research group in advance. Its main objective was to stimulate the sharing of experiences concerning factors that facilitate or hamper more home time and home deaths for patients with terminal disease and short life expectancy.

Four focus groups were conducted in an urban area of Norway: one with home care nurses, one with home care nurses and home care nursing assistants together, and two with GPs. Each focus group lasted from 80 to 100 min. The two focus groups with home care personnel took place in June and October 2015, whereas the two with GPs took place in May and June 2016. Local nursing managers recruited the nurse participants. The intention was to recruit GPs via the Local Medical Officer; however, this did not eventuate. We had to recruit them via the telephone: 17 GPs were contacted and eight agreed to participate. In total, 19 health professionals participated in the focus groups. Each focus group consisted of 4 to 6 participants. The GPs were recruited from different districts, whereas the nurses and nursing assistants were from one home care district.

The nurses were all experienced. On average, they had 24 years in clinical practice and 17 years in home care nursing. The age varied from 41 to 63 years, with an average of 52 years. Only three of them worked full-time, the rest worked part-time in positions, which varied from 54 to 80% of a full-time load. All Registered Nurses had further education and relevant competencies in home care nursing, palliative care, elderly care and health promotion (See Table 1). The home care nurses had recently attended a course in palliative care in their district. The collaboration with the patient’s GP was the responsibility of the home care nurses. The nurse assistants contributed to the primary care of the patients and assisted in contacting the next of kin.

**Table 1:** Participant characteristics – Home care nurses and nursing assistants

Profession	Age	Full-time / part-time?	Years of working experience as nurse / nurse assistant	Further education
RN Nurse 1	40–50	Part-time	20–25	Elderly and Aging
RN Nurse 2	40–50	Part-time	25–30	Home Care Nursing
RN Nurse 3	50–60	Part-time	35–40	Science in Nursing
RN Nurse 4	50–60	Full-time	10–15	Palliative Care Nursing
RN Nurse 5	50–60	Part-time	10–15	Palliative Care Nursing
RN Nurse 6	50–60	Part-time	25–30	Health promotion and Health prevention
RN Nurse 7	40–50	Full-time	15–20	Cancer Nursing
Nurse assistant 1	50–60	Full-time	10–15	None
Nurse assistant 2	50–60	Full-time	15–20	Dementia Care
Nurse assistant 3	60–70	Part-time	20–25	None
Nurse assistant 4	50–60	Part-time	25–30	Dementia Care

The GPs were all experienced doctors with an average of 24 years in professional practice. Their ages varied from 35 to 65 years, with an average of 56 years. All, except the youngest GP, were specialists in General Practice medicine (See Table 2).

**Table 2:** Participant characteristics – General Practitioners

Profession	Age	Full-time / part-time	Years of working experience as GP	Specialist competencies
GP 1	30–40	Full-time	1–5	Under specialisation
GP 2	40–50	Full-time	10–15	GP
GP 3	60–70	Full-time	25–30	GP
GP 4	60–70	Full-time	30–35	GP, Social Medicine
GP 5	60–70	Full-time	30–35	GP
GP 6	50–60	Full-time	25–30	GP
GP 7	60–70	Part-time	25–30	GP
GP 8	60–70	Part-time	30–35	GP

### Data collection

The four focus groups were conducted by two of the researchers. In the first focus group with the GPs, written notes were taken. These were subsequently used as the basis for the next focus group with the GPs. The other three focus groups were audiotaped and transcribed.

### Data analysis

The data were processed by systematic text condensation before analysis according to Malterud [27], which is a modification of Giorgi’s phenomenological analysis. Systematic text condensation encompasses thematic analysis of meaning and content of data across cases. It includes four steps of analysis: 1) *Total impression – from chaos to themes* - establishing a general impression of all the data from the focus groups with an open mind, looking for preliminary themes related to the participants´ experiences with palliative care in the home; 2) *Identifying and sorting meaning units – from themes to codes* – identifying and arranging meaning units by reviewing the text line by line, followed by coding of the meaning units related to the themes which emerged in the first step (A meaning unit is a text fragment including some information about the research question and may be coded under numerous code labels); 3) *Condensation – from code to meaning* – requiring systematic abstraction of meaning units within each of the code groups identified in the second step of the analysis. The empirical data are reduced to a decontextualized selection of meaning units sorted as thematic code groups. The coded units of meaning are condensed and organized into groups according to their code, sorting them into subgroups; and 4) *Synthesizing – from condensation to description and concepts* – reconceptualising the data by putting the pieces together. By synthesizing the contents of the condensed data, the description and concepts develop as an analytical text supported by citations, which can illustrate the research theme or finding.

### Approvals and ethical considerations

Ethics approval was received according to Norwegian research law and regulations. Each participant received written information about the research project in advance and provided informed consent before the focus groups were conducted. The research project was approved by the Data Protection Authority within the Norwegian Centre for Research Data (NSD) (Reg. no. 45256). The participants were informed both in writing and orally about confidentiality and anonymity of the data, and that at any stage they could withdraw from the study without any consequences.

## Results

In the current study, we identified the experiences of home care nurses and GPs of the conditions that facilitate or hamper more time at home and more home deaths for patients with terminal disease and short life expectancy. The results from the data analysis are divided into three different themes with 13 sub-themes altogether:

1. *The importance of a good start for the patient and family*
  - Establishing trust and safety for the patient and family
  - The important first days at home after discharge from hospital
  - Establishing collaboration between the home care nurses and the GP
  - Establishing safety for the patient and family by ensuring availability of the GP
  - The GP’s initial consultation in the patient’s home

2. *“Passing the baton” – the importance of collaboration across the health system*
  - Lack of collaboration with the hospital in the transfer of patients
  - Good support from the hospital Palliative Care Centre
  - The GP’s disconnection during hospitalization with consequences for the patient and family
  - A vulnerable gap in preparing the transfer of patients from the hospital
3. *Avoiding new hospitalization by establishing collaboration and competence within primary health care.*
  - Dialogue and interdependence between the GP and home care services
  - Competence, experience and counselling
  - Preparing for the end of the terminal stage for the patient and family – to be ahead
  - Tolerance, acceptance and flexibility within home care personnel

The themes and their sub-themes are defined below, with supporting data drawn verbatim (translated into English) from the focus groups and presented in italics.

### The importance of a good start for the patient and family

The home care nurses experienced that most of their patients in palliative care were transferred from hospital. However, some patients were already in home-based care with whom they had already established a close relationship, as well as with the family.

#### Establishing trust and safety for the patient and family

The nurses emphasized the importance of achieving a close and trusting collaboration with the patient and next of kin. They described how they prioritised these patients when establishing palliative care in the patient’s home. A primary group for the patient and family was established among the nurses in order to create more continuity and increase patient safety. The nurses underlined the importance of good collaboration from the very first day with the next of kin who had a key role in the success of home palliation. Empathy for, and time to listen to, the patient and family were considered essential. For reassurance, they gave the patient and next of kin the telephone number of the group and assured them of their availability 24/7.

*“It all depends on the next of kin for this to work. We cannot do the job unless the family is safe. Without the family, it is very difficult to succeed with palliative care at home.”* (Nurse)

#### The important first days at home after discharge from hospital

The home care nurses stressed the importance of the initial palliative care planning in the patient’s home following discharge from hospital in gaining the confidence of, and establishing the necessary safety for, the patient and the family. They emphasized good collaboration with the hospital as a critical factor to prepare for and obtain a good start. Consequently, they found it important to collect adequate information on the patient in advance in order to be prepared with the requisite medicines and medical equipment for the patient, such as a hospital bed, pain relief pump, professional displacement equipment, etc. This was considered important to maintain pain control, comfort, and quality care for the patient at home.

*“It is so important to have a good start. There are no practical things that paralyze more than lack of medications, displacement equipment, etc. (…) For if the start is chaos, then this chaos can easily continue. ”* (Nurse)

In general, the home care services selected an experienced nurse for the first meeting with the patient and next of kin in the patient’s home in order to gain confidence and ensure safety. To make sure that everything was properly set up, the first meeting often lasted one hour to fulfil the two key requirements: safety and trust. However, as they had many patients in the district to care for, there were not always adequate resources.

*“We need to get some extra time from the management on the first days. It’s a wish, but we do not always get it.”* (Nurse)

The family was often a great help in collecting medicines from the pharmacy or carrying out other practical errands. In recent years, all prescriptions in Norway have become electronic which has improved the daily administration of medications. Despite this, it sometimes takes several days to get the medicines from the pharmacist because they are not available locally.

#### Establishing collaboration between home care nurses and the GP

It was essential for the nurses to establish collaboration with the patient’s GP as soon as possible. They preferred to arrange a meeting at home with the patient, family, GP and nurse together. The GPs involved in the focus groups gave their cell phone number to the home care nurse when the life expectancy of the patient was short. The nurses often experienced good collaboration with the GPs, especially GPs from the local district whom they knew, and with whom they had regular contact. In their view, the GP’s involvement was fundamental for success.

*(…) “When the GP is not doing his or her job, it is simply not acceptable. We cannot take on their responsibility.”* (Nurse)

#### Establishing safety for the patient and family by ensuring availability of the GP

The GPs involved in the focus groups found it natural to give their cell phone number to the patient and family when there was short life expectancy for patients. The GPs said that they did not experience any misuse. According to the nurses, access to the GP’s cell phone made the patient and family feel safer and more confident. They believed that this strengthened the family’s ability to cope with the challenges of having to care for a very sick family member and avoid hospitalization. For these patients, the GPs set few limits with regard to working hours and medical support.

*“Once I had to take an emergency visit on Christmas Eve, where it was necessary to spend time at home in a family with a terminally ill patient who had a lot of pain and unrest. With conversation and drug titration, we managed to get the pain under control. (…) I could not let this occur during perhaps their last night at home together.”* (GP)

However, the GPs often sought agreement from the family that they would not call at night between 11 p.m. and 7 a.m. Home visits were made when needed, often in the afternoon after their office work had finished.

#### The GPs initial consultation in the patient’s home

Sometimes the GPs planned home visits together with the home care nurses in the initial phase. They found this useful with regard to responsibilities and further planning.

*“As a GP, you totally depend on good collaboration with home care nurses. (…) It works best when home care nurses are confident in their role and take clear responsibility.”* (GP)

The GPs who participated in the focus groups all favoured home visits. They mentioned that the difficult life and death topic was easier to address there. When life expectancy was short and little time was left, they also experienced that it was far more efficient to visit patients at home.

*“We become much closer to the patient at home where we can go into the kitchen and perhaps drink a cup of coffee together.”* (GP)

### “Passing the baton” – the importance of collaboration across the health system

The health care system in Norway is divided into specialized and primary health care which means that the medical responsibility of the hospital doctors and the GPs is separated. When the patient is hospitalized, the GP generally is not involved in medical treatment.

#### Lack of collaboration with the hospital in the transfer of patients

The home care nurses described how the hospital often promised more on behalf of the home care services than was realistic to achieve, which could often lead to unrealistic expectations from the patient and family. The nurses experienced that short hospital stays were the main focus of the hospital. In Norway, the principal policy is to have as few hospital beds as possible, based on calculations and historical data for optimum use. Early discharge is therefore stressed, putting pressure on doctors to make it possible to receive new patients. There is also a regulation which states that as soon as a hospital doctor declares that medical treatment is completed, the municipality has to take over the care of the patient. Otherwise, the municipality has to pay daily fines. According to the nurses, this often resulted in palliative care patients being discharged too early and being unprepared or with false expectations about what was possible for the home care nursing services to facilitate.

*“The hospital said that we (the home care nursing team) could be there all the time if needed. They give patients and next of kin unrealistic expectations. (…) We cannot be there 24 hours a day.”* (Nurse)

The GPs were concerned with the hospital’s responsibility and urged them to “pass the baton” to make discharge of the patient safer. They referred to the formal collaboration agreements between hospital and community care.

*“Friday is a very bad day for discharging palliative care patients (…) It is their responsibility, and it is the hospital's responsibility to make sure; it’s about passing the baton to the next doctor in a proper way.”* (GP)

The GPs experienced lack of contact when the palliative care patient was ready for discharge. They described the management in the municipality home care services being contacted by the hospital, but there was no protocol for contacting and establishing collaboration with the GP. Instead, what they did receive was an electronic medical report shortly after a hospital discharge, sometimes the next day.

#### Good support from the Hospital Palliative Care Centre

According to the GPs, the Palliative Care Centre was the only department in the hospital that worked together with the GPs as a team. The Palliative Care Centre contacted the GP as part of the routine procedure prior to the patient’s transfer, in order to establish the necessary collaboration. In general, the GP’s home visits needed to be planned up front, due to their already full schedule with other patients.

The home care nurses had good experiences and collaboration with the Palliative Care Centre, especially with their multidisciplinary ambulatory team. The nurses’ experience was that this team could compensate in cases where the GP was not sufficiently involved and sometimes they worked as professional support for both the GP and the home care nurse team.

*“The patient came home Friday and died the following Tuesday. He got urinary retention due to cancer prostate. I tried to insert a urinary catheter, but I did not succeed. The patient was in terrible pain. The GP came, but he failed too. He called the Palliative Ambulatory Team (…) and a doctor and a nurse came to the patient’s home. With a special urinary catheter, they succeeded. The patient calmed down and died just an hour or two afterwards in his own bed at home surrounded by his family”.* (Nurse)

#### The GPs’ disconnection during hospitalization with consequences for the patient and family

The GPs said that when one of their patients was hospitalized, they were rarely contacted by the hospital physicians about the patient’s medical situation or invited to discuss the medical treatment. They described their situation as being ‘disconnected’ from the medical treatment, and they urged establishing two-way communication with the hospital physicians, which they thought would benefit the patient and next of kin and increase patient safety.

The GPs often experienced having an important translator and liaison role between the patient and the hospital during medical treatment. The patients met many physicians and health professionals during their hospital stay, and the patients often found it confusing to understand the treatment initiated, etc. In the GPs’ view, it could be difficult for the patient to provide informed consent. The nurses reported similar experiences; they needed to explain the treatment, etc. in understandable terms. The GPs underlined that they generally knew their patients very well, and believed they played a central role in patient care.

*“Patients are in a phase where they are desperate; they meet so many new doctors. It allows me as a GP to become much more important for the patient in the hospital phase.*” (GP)

The GPs urged the hospital to build closer collaboration with the GPs in their routines for the benefit of palliative care patients. In their opinion, both medical and ethical dilemmas could have been solved for the better. Their experience was that the hospital did not even consider the GP’s knowledge of the patients. The GPs expressed that “overtreatment” and “cowboy medicine” could be reduced by involving them through the medical treatment process.

*“(…) I have known the patient for more than 30 years. Now she is 87 years old, suffering and multimorbid. (…) They start new treatment without even considering consulting me.”* (GP)

#### A vulnerable gap in preparing transfer of patients from hospital

The home care nurses described the procedures and experiences when a palliative care patient was discharged. The hospital contacted the local management in the Home Services of the appropriate district within the municipality. The management then communicated with the local district’s home care nurses who organized the service for the patient. They worked in different offices some miles away. The home care nurses rarely had direct contact with the hospital ward that was responsible for the discharge of the patient, despite this previously having been the practice. Instead, they received electronic information through the management. The nurses worried about patient safety, as they had often experienced that vital information was overlooked or lost during those steps.

### Avoiding new hospitalization by establishing collaboration and competence within primary health care

Collaboration, competence-building and flexibility *within* primary health care was emphasized by both the home-based nurses and the GPs.

#### Dialogue and interdependence between the GP and home care services

Sometimes the nurses struggled to get the GP involved in palliative care. It was a matter of the GP’s priorities as well as confidence in the nurses´ clinical judgement of the patient. The patient’s condition could deteriorate quite suddenly, and therefore it was of great importance that the nurses and GP established regular contact and confidence in each other and consequently were able to collaborate closely. If this did not occur, the risk of hospitalization increased.

*“If you don’t have a GP who is there for the palliative care patient, it’s hard to get the job done properly. Sadly, it often ends with death in the hospital. We are simply not able to manage it at home, because it’s not safe when the GP does not participate.”* (Nurse)

The communication between home-based nurses and GPs had improved by introducing a system of electronic messages. The nurses could write messages, which the GP could respond to during the day. Both home care nurses and GPs underlined that electronic communication with dialogue messages was a great improvement and increased the availability of both parties for the benefit of the patients.

#### Competence, experience and counselling

The GP often discovered that many home care nurses had expertise in palliative care with far more experience than they had with these patients. The GPs thought they should be more aware of this aspect in the collaboration.

*“My experience is that home care nurses are doing well in palliative care and are very familiar with handling pain relief pumps, far better than myself.*” (GP)

The GPs appreciated the dedication and engagement of the nurses to do their best for the patients and their next of kin. However, they did expect home care nurses to come forward and express clearly their expectations in the collaboration. The GPs experienced that when the home care nurse was uncertain, it required more of them. They wanted the nurses to have the confidence to come forward and express what they needed from the GP.

On the other hand, the GPs acknowledged that home care nurses had challenging work, and that it was important to have somebody to discuss complex and ethical matters. The GPs said they had little training in palliative care medicine. They highlighted the courses in palliative care medicine organised in collaboration with the Palliative Care Centre and the hospital. If possible, they preferred to have courses together with home-based nurses in their local districts.

The home care service had nurses with special competencies in palliative care in their team. The nurses emphasized the importance of clinical expertise and advanced experience in interpersonal and technical situations to create the necessary safety for palliative care patients and their families. They stressed that this was not an area for novices. The nurses who participated in the focus groups had many years of experience but expressed the need for increasing their competencies by gaining new knowledge. Relevant courses and meetings with professional thematic content and discussions were highly appreciated.

The nurses expressed a need for regular counselling. Intense working periods with palliative care patients could be quite exhausting and stressful both physically and mentally. Sharing experiences happened randomly when the nurses had time or felt it was necessary. However, they called for leaders to take greater responsibility for coordinating systematic mentoring and counselling for the nurses.

#### Preparing for the end of the terminal stage for the patient and family – to be ahead

Both nurses and doctors were aware of the vulnerability of the patient and family when the patient’s situation deteriorated. The nurses tried to be prepared for what was coming and to create a good environment for the patient in close collaboration with the family. At the final terminal stage, home care registered nurses, and not the nurse assistants, took care of the patient and family as they experienced that their competences were needed at all times. They wanted to create predictability for the patient and family and to practise flexibility according to the patient’s and family’s needs and wishes. Both GPs and nurses were readily available on the telephone.

“*The daughter of a dying mother could never forget how thankful she was when the nurses acted flexibly and said: “We will come back later, when you and your mother have finished your breakfast.” (…) Then you realize that such small things become so important to them.”* (Nurse)

The nurses were concerned about the families who had promised their loved ones that they could die at home, as the situation could change very quickly, and the patient’s situation could become unbearable. In those situations, they experienced that the hospital could be a better alternative for the patient and family and they needed to support them in that decision.

The nurses made great efforts to have the necessary medication available in time for relieving the most common symptoms in the terminal stage. It was important for the nurses that the GP prescribed the medication in a timely manner, as the patient’s situation could change very quickly. Without the requisite medicines, it often ended with hospitalization for the patient. A medical box, similar to that used in an English model, with the four most important medicines for symptom relief in the terminal stage (the ‘just-in-case box’), was introduced at the Palliative Care Centre and the Centre for Competence in Palliation consisting of Morphine, Midazolam, Haloperidol and Glycopyrron [2, 28]. The medical box was prescribed from the GP or Hospital Palliative Care Centre, which made it possible for home care nurses to control patient’s symptoms in the last terminal stage and avoid hospitalization.

#### Tolerance, acceptance and flexibility within home care personnel

The home care nurses had been a team for many years with little turnover and they highlighted the importance of good collaboration within their own group. They helped each other out, shared experiences and supported each other, which was essential to keep up the enthusiasm and pleasure in the work. Competence, clinical experience, palliative care experience, maturity, situation recognition and acceptance of each other’s different skills were highlighted.

They seemed to handle new technical equipment and procedures with great competence to ensure patient safety. They expressed having a good and supportive atmosphere and an ability to speak openly with each other.

*“We certainly have the will to help each other. (...) There is mutual understanding that those who work with palliative care patients and dying patients, they need time.* So, *we help each other.”* (Nurse)

The home care nurses practised flexibility in the shift roster when it came to situations where they needed more time in the palliative care patient’s home. They could not always get hold of an extra nurse. They relieved each other of different additional tasks and assignments during the day to make it possible for the nurses involved to handle the situation without having to think about every task that awaited them. They had a kind of “silent agreement”. The flexibility made it easier for them to cope with challenging situations, which could occur quite suddenly.

*“I went overtime and started with morphine, satisfied since we had “saved the situation” (…) Then you cannot leave your job. You simply must stay at work an extra couple of hours.”* (Nurse)

## Discussion

Several studies have shown connections between individual, collaborative and organizational aspects of the palliative care services and the rates of home deaths [11, 19]. Early referral from hospital to home-based care and multidisciplinary palliative home care teams are essential findings [8]. In our study, we wanted to achieve more insight, through home care nurses and GPs, of conditions that that facilitate or hamper more time at home and more home deaths for patients with terminal disease and short life expectancy.

### The importance of a good start

The nurses underlined that it was essential to establish close collaboration with the patient and family early as well as emotional support for the family who took on such a great responsibility. The primary group contributed to more continuity and safety stressing the importance of not leaving the professional responsibility of palliative care to the family. This is in line with other studies which have identified the significance of close and personal collaboration and contact with the family in palliative home-based care [29, 30].

The nurses emphasized that the collaboration meeting between the patient, next of kin, GP and home care nurse ought to take place as soon at the patient arrives home. This is essential to secure a good start. This meeting needs a fixed agenda. It must be clarified if the patient really wants to stay at home as long as possible and even die there. The capacity and support from the next of kin is vital. Moreover, the initial preparation dialogue should focus on what kind of complexity they can endure at home [31].

Furthermore, it must be clarified as to what kind of medical complications might make the patient’s family think it would be better for the patient to be in hospital. They need to be reassured that their decision can be changed. The dialogue needs to focus on both their current worries, as well as the challenges ahead.

It is important that the nurse has the necessary experience and skill to participate in these meetings [32]. To succeed with a good start, a very competent home care nurse must be present at the first meeting. The main purpose is to create an atmosphere of trust and to reduce anxiety. The home care nurses must give priority to the family and be sure there is enough time to do so. Trust from both the patient and the next of kin is the goal. The nurses in our study stressed that if home-based palliative care started in chaos, the chaos was likely to continue, and they expressed that they would never leave the family unless the family knew who and where to call 24/7.

Our study underlines the importance of creating safety at home for the patient and family by ensuring good planning in advance, together with the hospital, when the patient is still hospitalized. That includes medications and medical equipment necessary for pain control, and other relief for the patient at home. Such practical aspects of discharge planning and coordination were also emphasized by O’Brien & Jack [21] as pivotal. Other studies have found that advance care planning with respect to the place of death reduced the time in hospital in the patient’s last year [33] and that patients who had issued directives in advance received care that was strongly associated with their preferences [34]. The participants in our study stressed the importance of establishing a collaboration between the patient’s GP and home care nurses. GP home visits create openings for closer collaboration with the patient and family as well as the opportunity to talk about the life and death topic [31]. Another study indicated that GP home visits in palliative care had an important impact on the patient staying and dying at home [35].

### Passing the baton between the hospital and home-based palliative care

In Norway, the health care services are regulated by different laws and authorities that can present challenges to collaboration. The specialist palliative care services in the hospitals are administered by one of the four Regional Health Authorities. Home care nurses and self-employed GPs in the primary health care sector are administered by the 428 local municipalities.

Our findings revealed challenges with the continuity of palliative care between the hospitals and the primary health care sector. The GPs were rarely contacted prior to the patient’s discharge from hospital, despite having in-depth knowledge of the patient. They did not feel that their knowledge was acknowledged by the hospital doctors concerning further treatment plans for the patient. The GPs were left with a sense of being disconnected from the medical treatment. They were concerned about patient safety and urged the hospital physicians to establish two-way communication. Instead, the GPs received a final medical report. Although the municipal home care services were contacted by the hospital prior to the patient’s discharge, either by phone or via a written report, the nurses expressed that the planning and preparation by the hospital was often inadequate.

One problem is that information between the hospital and those responsible for palliative care in the primary health care sector generally goes to the municipality’s health care service management rather than directly to the GP or primary home care nurse. Even when given the expectation, participants could not always trust that prescriptions, medicines etc. were in order. The most difficult transfer day in the week was Friday, due to the difficulties of adequate supply of medicines during the weekend, as vital information was often missing upfront. Normally, the patient’s GP was not available in the weekends. Our findings emphasize the need for safer discharge routines when hospitals transfer palliative care patients to their homes. Electronic two-way communication between hospital doctors and GPs would increase patient safety.

Sometimes there seemed to be a huge difference between what patients and families had been promised at the hospital and the reality of what home care nurses were able to provide. This caused insecurity and a drop in confidence in the patient and next of kin. According to the home care nurses and the GPs, the hospital should pass the baton in a proper and safe way that is better than that seen in current practice.

The palliative care patients are in vulnerable situations that require special arrangements and a dependence on continuous exchange of knowledge, information and decisions. Several earlier studies have emphasized the importance of early referral and knowledge exchange between the hospital and those delivering palliative care in the primary health care context [36–38]. Earlier involvement and knowledge exchange allows relationships to be built and trust to be developed in the care and treatment planning processes between the collaborators. Such contact promotes safety for the patients and prevents crises from occurring. Some studies have found that the next of kin has a key role [11, 19, 32]. Our findings indicate that without a family member to take responsibility, it is almost impossible to achieve good palliative care at home.

### Avoiding hospitalization by establishing safety and competence

In order to avoid hospitalization in the terminal stage of life, our research shows that dialogue and interdependence between nurses and GPs is crucial and prevents unnecessary hospitalization. The nurses appreciated good collaboration with GPs. If the GP does not take an active part, the patient and family is left in a more vulnerable situation. Although the Palliative Care Centre at the hospital is available in some areas, it is necessary to team up with a GP in order to succeed in the delivery of good palliative care.

The health professionals’ competence and experience in palliative care are essential. Our study revealed the need for courses in palliative care that include a focus on collaboration across all palliative care providers. This is in line with Smith and Porock [36] who found that training and educational interventions were of crucial importance to improve knowledge and practical competence in palliative care in the community. Both GPs and nurses in this study reported the need to work together, “shoulder to shoulder”, and they appeared dedicated to their palliative care work. According to Levine et al. [39] and O’Mahony et al. [40], palliative care work required the health professionals involved to take care of each other.

However, the nurses reported that the GPs did not always trust their clinical judgement. The patient’s condition could alter quickly and consequently they needed to be prepared; for instance, in the choice of medication or subcutaneous administration instead of tablets if swallowing problems should occur. Difficulties in establishing collaboration with the GP may be due to GPs’ uncertainty caused by their lack of experience with palliative care patients. However, further research into this aspect is needed.

Some GPs emphasized that nurses should be more outspoken in communicating their understanding of the patients´ needs. Instead of expressing despair, they urged nurses to be more assertive and express clearly what they expected from the GP. This feature may be an aspect of health professional hierarchy where nurses have often felt oppressed by doctors [41].

The nurses and GPs in our study stressed the importance of close collaboration with, and emotional support for, the patient and family and the need for flexibility and availability especially in the last part of the terminal stage, which is in accordance with other research in the field [29, 30, 42]. Even if they were dedicated to palliative care, they experienced that the family was fundamental in achieving more time at home for the patient and eventually the chance for the patient to die at home. The use of the medical box for symptom relief was essential and avoided some unnecessary hospitalizations. Instead, they managed to control the symptoms and the patient could die peacefully at home [28].

On the other hand, the nurses reported that patient’s condition could worsen very quickly, and the family was not always capable of handling the situation, as it could become unbearable. They found it important to support the family members to change their minds even if they had promised their loved one to die at home. Supporting the family to balance between their needs, wishes and resources has been found to be critical in home-based palliative care [29, 42]. The dying patient and family need reassurance from trusted professionals with experience in palliative care [43].

This study highlights the need for flexibility in shift plans and working lists as well as understanding within the nursing group. The nurses knew each other well and could rely on each other’s competencies. When the nurses realized that the death of a patient was imminent, they practiced flexibility in the shift plan. They had a kind of “silent agreement” to handle some of the tasks of the nurses involved so that they could stay longer with their patient. Without this flexibility, these patients would often be sent to the hospital or emergency unit.

The nurses missed being mentored and having the opportunity to talk about difficult situations and ethical dilemmas in an organised way. Working with palliative care patients and their families is demanding in many ways. It is well known that exhaustion and stress is a huge concern for palliative care health professionals [40, 44]. Supportive mentoring on a regular basis could reduce and prevent distress [39].

### Methodological considerations

Gaining perspectives from both home care nurses and GPs could be considered a strength. Our participants were particularly experienced and quite dedicated, which might reduce their representativeness. Our study focused on the professionals working with palliative care in primary health care, not professionals working in hospitals. Hence, there is a limitation as the voice of doctors and nurses in hospitals has not been heard regarding the transfer of the palliative care patient from hospital. Because of our context, the findings are probably more relevant to cities, rather than rural areas. However, it was not the aim of the study to investigate this issue in different geographical contexts. Further research into this aspect could be useful.

## Conclusion

This study demonstrates that optimum palliative care at home depends on close collaboration and dialogue between the patient, family, home care nurses and general practitioner. Our findings emphasize the need for safer discharge and planning procedures when hospitals transfer palliative care patients to their homes. A good start is crucial for a good result. The initial collaboration meeting at home between the patient, next of kin, general practitioner, and home care nurse requires a fixed agenda. An interesting and thought-provoking finding was that the general practitioner was rarely contacted during their patient’s hospitalization and prior to discharge despite having in-depth knowledge of their patient’s medical history, which if sought, could benefit the patient and the next of kin. This ‘disconnection’ with the patient’s general practitioner might reduce patient safety. Both general practitioners and home care nurses appeared to be dedicated to their palliative care work. They emphasized the need to work together, “shoulder to shoulder”. However, they experienced that the family was fundamental in gaining more time at home for the patient and creating the chance for the patient to eventually die at home. Our study emphasizes that home-based palliative care demands experience and competence as well as regular supportive mentoring.

## Abbreviations

GP: General practitioner
NSD: Norwegian Centre for Research Data
RN: Registered nurse
